# Research on Machining Parameter Optimization and an Electrode Wear Compensation Method of Microgroove Micro-EDM

**DOI:** 10.3390/mi16040481

**Published:** 2025-04-18

**Authors:** Xiaodong Zhang, Wentong Zhang, Peng Yu, Yiquan Li

**Affiliations:** Ministry of Education Key Laboratory for Cross-Scale Micro and Nano Manufacturing, Changchun University of Science and Technology, 7089 Weixing Road, Chaoyang District, Changchun 130022, China; dushu6688@foxmail.com (X.Z.); 15865001927@163.com (W.Z.)

**Keywords:** micro-EDM, electrode wear compensation, orthogonal experiment, grey relational analysis, artificial neural network, image processing technology

## Abstract

In the process of micro-EDM, tool electrode wear is inevitable, especially for complex three-dimensional cavities or microgroove structures. Tool electrode wear accumulates during machining, which will finally affect machining accuracy and machining quality. It is necessary to reduce electrode wear and compensate it through micro-EDM. Therefore, based on an established L27 orthogonal experiment, this paper uses the grey relational analysis (GRA) method to realize multi-objective optimization of machining time and electrode wear, so as to achieve the shortest machining time and the minimum electrode wear during machining under the optimal machining parameter combination. Then, the orthogonal experiment results are used as dataset of artificial neural networks (ANNs), and an ANN prediction model is established. Combined with image processing technology, the bottom profile of the machined microgroove is extracted and then an electrode axial wear compensation equation is fitted, and a fixed-length nonlinear compensation method for electrode axial wear is proposed. Finally, the GRA optimal experiment shows that machining time, electrode axial wear and radial wear are reduced by 13.89%, 3.31%, and 10.80%, respectively, compared with the H17 orthogonal experiment with the largest grey relational grade. For the study of electrode axial wear compensation methods, the consistency of the depth and width of the machined microgroove structure with compensation is significantly better than that of the microgroove structure without compensation. This result shows that the proposed fixed-length nonlinear compensation method can effectively compensate electrode axial wear in micro-EDM and improve machining quality to a certain extent.

## 1. Introduction

With the rapid development of science and technology, modern industry puts forward higher requirements for the machining accuracy of micro parts [1,2,3,4]. Because of its good electrical conductivity, thermal conductivity, wear resistance and other unique properties, copper has become an important material for manufacturing precision micro parts, and is widely used in aerospace, precision instruments, and other fields. Among them, microgroove machining is an important part of micropart manufacturing, which broadens the application of micro parts [5,6,7,8,9]. Among the non-traditional machining technologies, micro-EDM is one of the most suitable methods for machining microgrooves [10] because it is not limited by the hardness and strength of materials and does not need to contact the workpiece directly [11]. The principle of micro-EDM is to use a pulse spark discharge between the tool electrode and the workpiece to locally and instantaneously remove material at a high temperature. However, the removal of material and tool electrode wear occur at the same time. Electrode wear will affect machining accuracy and machining quality, which seriously restricts the development and application in micro-EDM [12,13,14,15], especially for three-dimensional cavities or microgroove machining. Therefore, it is particularly critical to reduce electrode wear or to use corresponding methods to compensate for electrode wear during machining.

For the study of electrode wear compensation, researchers have proposed various effective models. In the 1990s, Professor Yu and Masuzawa [16] successfully processed a micro-car model using a simple-shaped micro-electrode based on the equal-wear theory. Its size was 500 μm in length, 300 μm in width, and 200 μm in height, creating a new field of micro-EDM with a micro-three-dimensional structure. Aliyu et al. [17] from Yamagata University proposed an on-line detection and compensation method based on an optical system. The CCD imaging system and optical sensor were used to monitor the changes in the electrode size and shape in real time during machining, and the corresponding compensation was carried out. Kaneko and Tsuchiya [18] Yu and Rajurkar [19] processed a micro three-dimensional cavity based on a uniform wear method of the CAD/CAM electrode, but the bottom surface of machined micro three-dimensional cavity was poor, which affected machining accuracy. Yu and Kozak [20] used a CAD/CAM system to research a method based on fixed plane reference compensation, and generated electrode motion trajectory milling based on electrode wear compensation in micro-EDM and successfully machined 100 μm micro-hemisphere structures on metal platinum material. Hang and Gao [21] proposed a new method of tool electrode spiral motion feed combined with fixed reference axial compensation, which not only promotes the discharge of debris in micro-hole machining but also improves machining quality. Gou and Lai [22] monitored the number of discharges during machining with Labview software (2019), established an empirical relationship between the number of discharges and electrode wear and proposed an electrode wear compensation method based on normal discharge and uniform wear. The experimental results showed that this compensation method can produce a microgroove structure with certain accuracy.

In this paper, to reduce electrode wear in micro-EDM and compensate for it, the multi-objective optimization of machining time and electrode wear was realized by the GRA method on the basis of an orthogonal experiment. And then a combined artificial neural network with image processing technology and a fixed-length nonlinear compensation method for electrode axial wear is proposed. And the study aimed to verify whether the proposed compensation method has certain compensation accuracy and rationality by experiment.

## 2. Experiment Platform and Method

### 2.1. Experiment Platform

**Figure 1:**
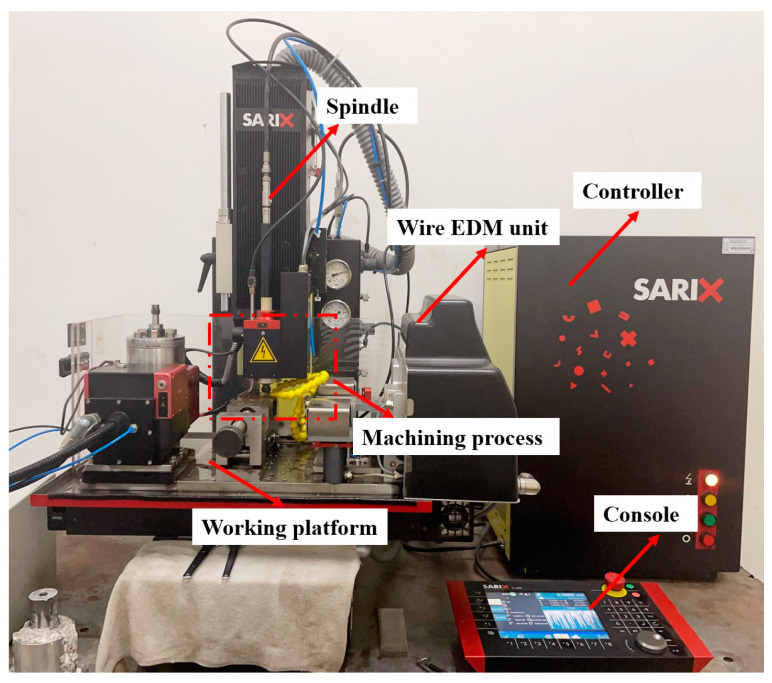
SX-100HPM EDM equipment.

The micro-EDM equipment was SX-100HPM (SARIX SA, Sant’Antonino, Switzerland), as shown in Figure 1, and the maximum travel distances of this equipment are 250 mm (X-axis), 150 mm (Y-axis), and 150 mm (Z-axis). The positioning accuracy of tool spindle is ±2 μm, and the motion’s resolution is 0.1 μm. The tool electrode has a diameter of φ250 μm and is made of carbide material, while the workpiece material is copper, with EDM oil as the working fluid.

The tool electrode is made of φ250 μm cemented carbide material, and the workpiece material is copper. Its performance is shown in Table 1.

In the process of machining, EDM fluid serves to reduce heat generation and effectively flushes the generated products of galvanic corrosion out from the machining area in a timely manner, keeping discharge gap in a stable state. Therefore, the choice of EDM fluid has a greater impact on the machining performance [23], and finally selected EDM oil as the working fluid, whose performance is shown in Table 2.

### 2.2. Grey Relational Analysis

Grey relational analysis (GRA) is a statistical analysis method used to evaluate the relationships among various factors in a model. In this paper, GRA is used to analyze and select the optimal level of influence factors, so as to realize shorter machining time and smaller electrode axial and radial wear in micro-EDM [24,25,26]. The GRA method follows these steps:

Step 1. The original data are divided into the comparison sequence and the reference sequence. The comparison sequence can reflect data sequence of system behaviour characteristics, similar to dependent variable *Y*, recorded as $Y={\left[y_{1},y_{2},\cdots ,y_{n}\right]}^{T}$. The comparison sequence is composed of factors that affect the behaviour of the system, similar to independent variable X, recorded as $x_{nm}=\left[\begin{array}{cccc} x_{11} & x_{12} & \cdots & x_{1m}\\ x_{21} & x_{22} & \cdots & x_{2m}\\ \vdots & \vdots & \ddots & \vdots \\ x_{n1} & x_{n2} & \cdots & x_{nm} \end{array}\right]$. The L27(3^5^) orthogonal experiment designed in this paper is used as the comparison sequence, and performance indicator results (machining time, electrode axial and radial wear) in micro-EDM are as the reference sequence for the following calculation and analysis.

Step 2. Normalize experimental comparison sequence and reference sequence in Equation (1) and Equation (2), respectively.

(1) $$\tilde{x_{i}}(k)=\frac{x_{i}(k)-\underset{k}{\min x_{i}(k)}}{\underset{k}{\max x_{i}(k)}-\underset{k}{\min x_{i}(k)}}$$

(2) $$\tilde{y_{i}}(k)=\frac{y_{i}(k)-\underset{k}{\min y_{i}(k)}}{\underset{k}{\max y_{i}(k)}-\underset{k}{\min y_{i}(k)}}$$

Step 3. Calculate grey relational coefficient in Equation (4).

(3) $$\xi_{i}(k)=\frac{N+\rho M}{\Delta y_{i}(k)+\rho M}$$

Among them, $N=\underset{i}{\min}\underset{k}{\min}|x_{0}(k)-x_{i}(k)|$, $M=\underset{i}{\max}\underset{k}{\max}|x_{0}(k)-x_{i}(k)|$. ρ is the resolution coefficient, its value range is within (0, 1), and the general value is 0.5.

Step 4. Calculate grey relational grade in Equation (4).

(4) $$\gamma_{i}=\frac{1}{n}\sum_{k=1}^{n}\xi_{i}(k)=\frac{1}{n}\sum_{k=1}^{n}y(x_{0}(k),x_{i}(k))$$

### 2.3. Artificial Neural Network

**Figure 2:**
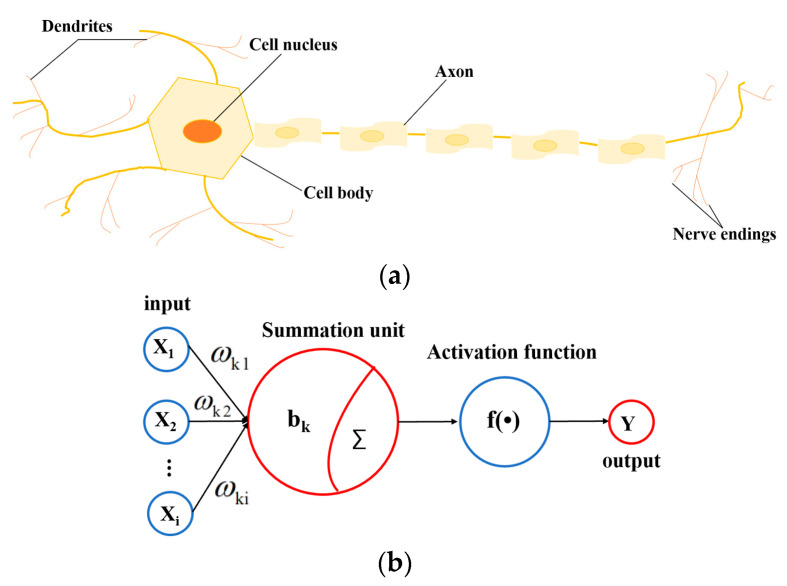
(**a**) A neuron cell; (**b**) ANN basic structure.

**Figure 3:**
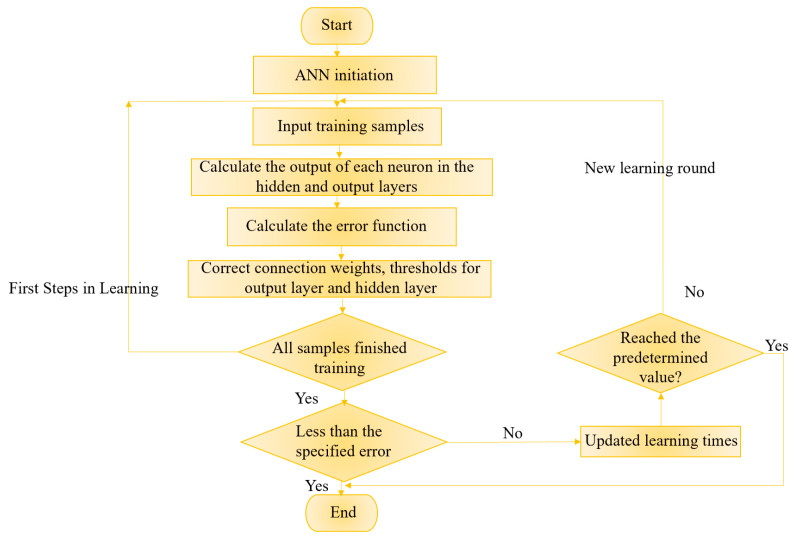
ANN learning and training process.

Artificial neural network (ANN) is a kind of information processing technology similar to human nervous system [26,27]. The neural network is mainly to abstract new functions of human brain and establish artificial neurons, which are connected according to a certain topological structure by simulating biological neural networks. As shown in Figure 2a, it is a simple neuron cell, which is composed of axons, dendrites and cell bodies, and relies on synapses to connect neurons. The transmitted signal travels from the axon of one neuron to the dendrites of another neuron, and this process known as forward propagation, which does not allow reverse propagation. Therefore, the neural network can be regarded as a directed graph, and connections between nodes are directed [27,28]. Many individual neurons are interconnected to form a neural network. This neural network processes input data by adjusting the weights between neurons, and changes its structure according to external information, enabling it to solve practical problems. The basic structure of ANN is shown in Figure 2b, which includes input, summation unit, activation function, and output. Each layer is interconnected by a large number of neurons. According to the external information, these neurons constantly adjust connection weight and threshold b_k_ to make error range of ANN training results meet the specific requirements. Figure 3 shows the flow chart of ANN learning and training process.

### 2.4. Image Processing Technology

Image processing technology first converts the input microgroove bottom contour image into a binary image through grayscale threshold segmentation, which is one of the most commonly used image segmentation methods. It compares greyscale value of each pixel with a threshold value and classifies pixels according to whether the threshold value is exceeded. The resulting segmented image is called a binary image. And then the edges of the binary image are detected and extracted. There are many operators for image edge detection and contour extraction. The common first-order operators include Robert operator, Sobel operator, Prewitt operator, Canny operator, and second-order Laplacian operator. The Robert operator [28,29] is suitable for processing steep low-noise images. The Sobel operator [29,30] and the Prewitt operator [30,31] are more accurate for edge positioning and are mostly used to process images with grey gradient and more noise. The Laplacian operator [31,32,33] is very sensitive to noise in an image, and it is seldom used in edge detection directly. The Canny operator is the most effective edge detection method, which can effectively reduce noise interference and detect real weak edges [33,34]. Therefore, the Canny operator is used to extract the bottom contour of microgroove in this paper.

## 3. Orthogonal Experiment and GRA Multi-Objective Optimization

### 3.1. Orthogonal Experiment Design and Result Analysis

In this paper, machining time, as well as electrode axial and radial wear, are selected as machining performance indicators in micro-EDM. After conducting a series of actual machining experiments with different influence factors, the electrode axial and radial wear can be measured. Due to electrode radial wear along its length direction of electrode is generally consistent in micro-EDM. On the premise of ensuring that the service life of equipment is not damaged and stable machining is carried out, three different levels of *I_p_*, *U_p_*, *f_p_*, *w_p_*, and *Rot* are selected, as shown in Table 3, and an L27 orthogonal experiment is designed. The orthogonal experiment table and the machining results, after taking three average values, they are recorded in Table 4.

**Figure 4:**
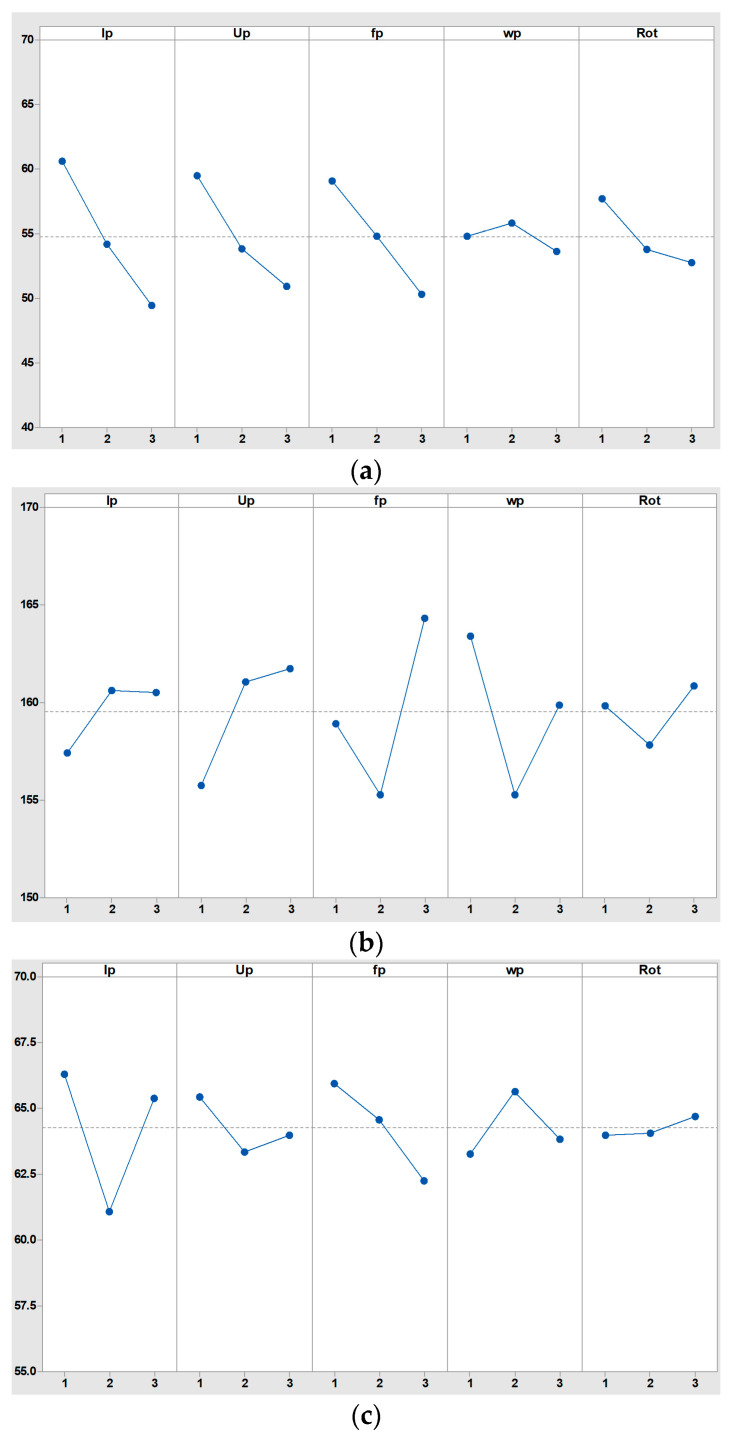
Main effect plot for machining performance indicators. (**a**) Main effect plot for machining time; (**b**) main effect plot for electrode axial wear; (**c**) main effect plot for electrode radial wear.

The results of the 27 orthogonal experiment results are analyzed, and the main effect plot for each machining performance indicator is drawn, as shown in Figure 4.

It can be seen from the main effect plot for machining time (Figure 4a) that when peak current *I_p_* is 35 Index, peak voltage *U_p_* is 110 V, pulse frequency *f_p_* is 130 kHz, pulse width *w_p_* is 5 μs, and spindle speed *Rot* is 800 rpm, the machining time for microgroove in micro-EDM is the shortest. And the influence of *I_p_*, *U_p,_* and *f_p_* on machining time is greater than that of *w_p_* and *Rot.* From Figure 4b, it can be seen that when *I_p_* is 25 Index, *U_p_* is 90 V, *f_p_* is 120 kHz, *w_p_* is 4μs, and *Rot* is 700 rpm, electrode axial wear for microgroove in micro-EDM is the smallest. From Figure 4c, it can be seen that when *I_p_* is 30 Index, *U_p_* is 100 V, *f_p_* is 130 kHz, *w_p_* is 4μs, and *Rot* is 600 rpm, electrode radial wear for microgroove in micro-EDM is the smallest. And the influence of non-electric parameter *Rot* on electrode wear is less than that of electric parameters *I_p_*, *U_p_*, *f_p_,* and *w_p_*.

### 3.2. GRA Multi-Objective Optimization

Through the main effect plot for orthogonal experiment results, it can be concluded that machining parameters, including peak current (*I_p_*), peak voltage (*U_p_*), pulse frequency (*f_p_*), pulse width (*w_p_*) and spindle speed (*Rot*), have different effects on machining time, electrode axial wear, and electrode radial wear. Additionally, the combination of optimal machining parameters under the single-objective optimization is also different. Therefore, this paper uses the grey relational analysis method to optimize machining time and electrode wear at the same time. According to Equation (4), the grey relational grade of the L27(3^5^) orthogonal experiment can be calculated and sorted, as shown in Table 5.

To achieve the simultaneous optimization of multi-performance indicators, the mean grey relational grade of each machining parameter and each level is obtained, as shown in Table 6.

The larger the range is, the greater the influence of machining parameters on multi-performance indicators is. It can be seen from Table 6 that *I_p_* has the greatest influence on multi-performance indicators, and the smallest is *w_p_*. The order of influence factors degree is *I_p_* > *f_p_* > *Rot* > *U_p_* > *w_p_*. The larger the grey relational grade of each factor and level, the more consistent the change trend between the comparison sequence and the reference sequence, including better performance of multiple indicators. It is found that when *I_p_* = 30 Index, *U_p_* = 100 V, *f_p_* = 120 kHz, *w_p_* = 4 μs, and *Rot* = 700 rpm, the microgroove micro-EDM achieves the shortest machining time and the smallest electrode axial and radial wear from Table 6.

From the L27 orthogonal experiment, it is observed that machining time for H17 is 55.87 s, electrode axial wear is 155.43 μm, and electrode radial wear is 62.24 μm. While the actual results in micro-EDM of machining time is 48.11 s, electrode axial wear is 150.29 μm, and electrode radial wear is 55.52 μm. Compared to the H17 orthogonal experiment, which has the largest grey relational grade, the GRA optimal experiment shows that machining time, electrode axial wear and radial wear are reduced by 13.89%, 3.31%, and 10.80%, respectively.

## 4. Electrode Axial Wear Compensation Experiment

**Figure 5:**
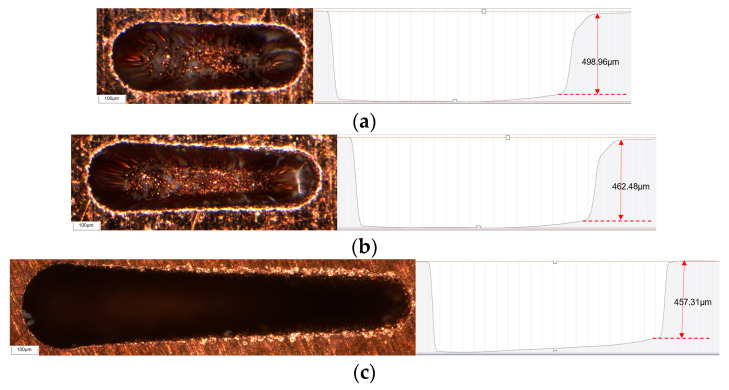
Microgroove morphology and depth with different lengths under GRA optimal experiment. (**a**) L = 510 μm; (**b**) L = 700 μm; (**c**) L = 1200 μm.

Under the combination of GRA optimization parameters, the same feed depth is set, and only the length of microgroove machining is changed, and an experimental study of microgroove micro-EDM is carried out. As shown in Figure 5, when the machining length (L) is set to 510 μm, 700 μm, and 1200 μm, the morphology and depth of the microgroove are machined. It can be seen that as the microgroove machining length increases, the electrode wear also increases, resulting in poor consistency in the depth and width of the machined microgroove, which negatively affects the accuracy and quality of the micro-EDM process. Therefore, compensating for electrode axial wear during the machining process is essential.

**Figure 6:**
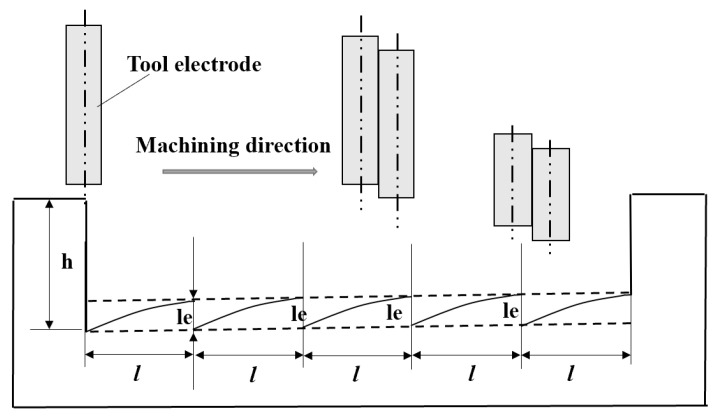
Fixed-length compensation schematic diagram.

Aiming to address the problem of electrode axial wear in micro-EDM, many researchers have proposed compensation methods. Among them, Pei Jingyu et al. from Shanghai Jiaotong University studied the fixed-length compensation method. The schematic diagram of compensation principle is shown in Figure 6. The electrode wear compensation is a fixed value under a certain machining length (L). However, there is no linear relationship between electrode axial wear and machining length, meaning there is no linear relationship between the change in the bottom profile and the machining length. If the fixed-length compensation method is adopted, the bottom surface of the machining cavity will appear a serrated shape, which will affect machining accuracy. Based on this defect, this paper improves the fixed-length compensation method and proposes a fixed-length nonlinear compensation method for electrode axial wear, utilizing image processing technology and an ANN prediction model. Image processing technology is used to obtain the theoretical electrode wear compensation path, while the ANN model is used to predict electrode axial wear along the machining compensation path.

**Figure 7:**
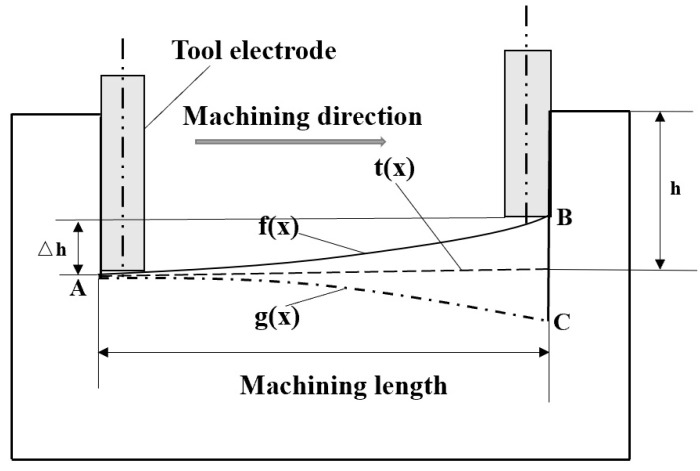
Electrode wear diagram in microgroove micro-EDM.

As shown in Figure 7, assuming the initial surface level of the workpiece (all subsequent research hypotheses follow this premise), when micro-EDM is used to machine the microgroove, the machining direction is from A to B, with a total length of L and an actual machining depth of h. Tool electrode programming machining trajectory is denoted as t(x). Due to electrode axial wear, the bottom profile curve of the machined microgroove is f(x). If the bottom profile of the machined microgroove is t(x), then g(x) is required as the theoretical electrode compensation path, and f(x) and g(x) are symmetrical about t(x). Considering that there will also be electrode axial wear △z during the process of micro-EDM compensation according to the theoretical compensation path, the actual electrode compensation amount should be g(x) +△z.

### 4.1. ANN Prediction Model Establishment

**Figure 8:**
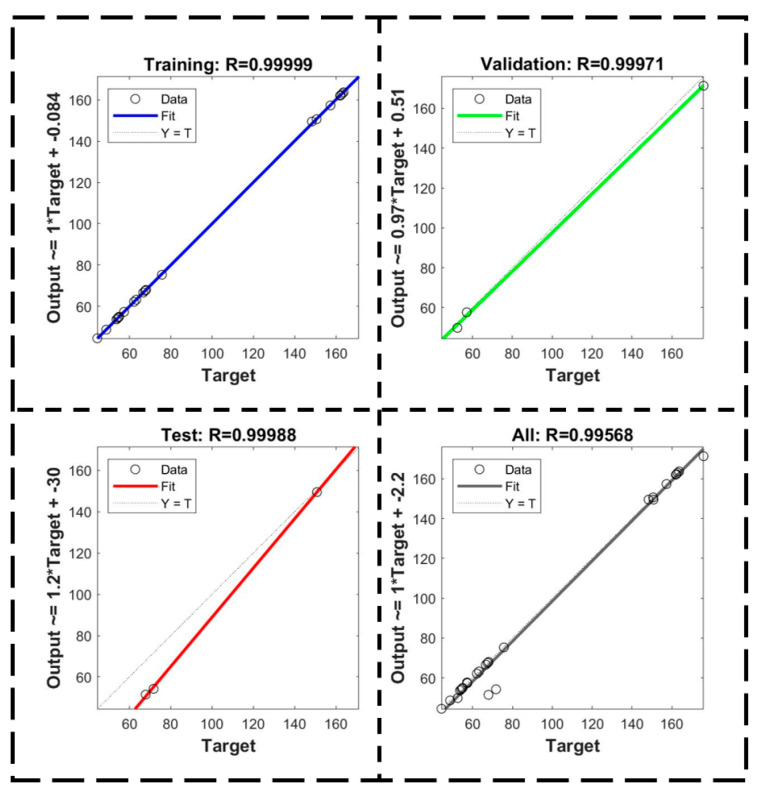
ANN model data training diagram.

In this paper, the input layer is composed of seven neurons, which are *I_p_*, *U_p_*, *f_p_, w_p_*, and *Rot*, machining length L, and feed depth H. The output layer is composed of three neurons, which are machining time, electrode axial wear, and radial wear, respectively. The number of hidden layers is a single layer, and a three-layer of ANN with seven inputs and three outputs is established. The number of ANN model’s training interaction is set to 1000, the learning rate is 0.01, and the minimum training error target is 0.001. And activation functions of hidden layer and output layer both select tanh function, and the Levenberg–Marquardt algorithm is selected as the training function for error signal backpropagation. The prediction accuracy (R) of the trained ANN can reach 0.99568, as shown in Figure 8.

To verify the feasibility and accuracy of the ANN prediction model and whether there is over-fitting phenomenon in this prediction model, the combination of parameters that are not available in the L27 orthogonal experiment is predicted. Specifically, the GRA optimization parameters are used for verification. Then, the prediction value of machining performance is compared with the actual value obtained from the GRA optimal experiment. The predicted value for machining time, electrode axial, and radial wear is 48.61 s, 150.76 μm, and 55.42 μm, respectively.

### 4.2. Image Processing Technology Analysis

**Figure 9:**
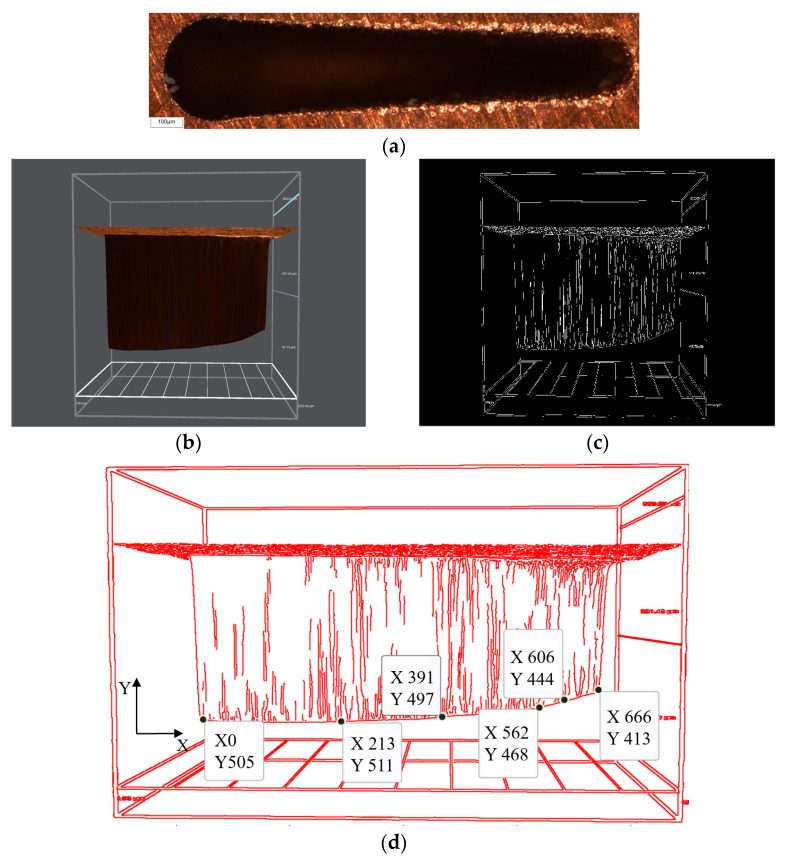
(**a**) Microgroove morphology without compensation; (**b**) Microgroove profile without compensation; (**c**) The binary image of profile; (**d**) The coordinates of microgroove bottom surface contour change.

Under the GRA optimization parameters, a feed depth of H = 600 μm and a machining length of *L* = 1200 μm are set for machining the microgroove. The microgroove morphology is shown in Figure 9a. The contour of the microgroove bottom surface is extracted and transformed into a binary image, and then the coordinate points of the microgroove’s bottom surface contour are extracted, as shown in Figure 9b,c.

In Figure 9d, X represents the length direction of the microgroove machining, and Y represents the depth direction. The microgroove with a length of L = 1200 μm is machined, and the X coordinate is transformed from 0 to 666. The Y coordinate from 0 to 505 represents the microgroove depth h in the actual machining process.The depth h is measured to be 457.31 μm by using an ultra-depth-of-field optical microscope. From this, the actual length or depth corresponding to each unit X or Y can be gotten. Through the corresponding relationship between f(x) and g(x), the extracted coordinate points of microgroove bottom contour are converted into coordinate points of the theoretical electrode compensation path, and then the curve equation of the theoretical electrode compensation path is obtained by nonlinear fitting using Matlab software (2019a). Because the electrode wear in micro-EDM is nonlinear, the commonly used function forms of electrode wear nonlinear fitted equation are the following three types [34,35]:

1. Exponential function

(5) $$y=ae^{bx}$$

2. Power function

(6) $$y=ax^{b}$$

3. Polynomial function

(7) $$y=ax^{2}+bx+c$$

These three nonlinear functions are used to fit the theoretical electrode compensation path curve based on the existing coordinate points, and the goodness of fit for each nonlinear function form is analyzed, including R^2^ (coefficient of determination), adj R^2^ (adjusted coefficient of determination), and the mean square error (RMSE), as shown in Table 7. The coefficient of determination R^2^ is in range of (0, 1). The larger the R^2^, the better the fitted. When R^2^ is less than 0, the effect of fitted nonlinear equation is very poor, and Adj R^2^ and R^2^ have similar significance. If R^2^ is negative, it further indicates that the fitted model fails to effectively represent the data, even worse than a simple mean value model. The root mean square error (RMSE) is used to measure the deviation between the predicted values of the model and the actual observed values. The smaller the RMSE, the better the model fitted. The R^2^ of the exponential function selected in this manuscript for curve fitting the bottom contour change can reach 0.9943, the adj R^2^ can reach 0.9924, and RMSE is 0.0080, which is close to 0. This indicates that the goodness of fit under this function form has reached the actual demand, and there is no need to complicate the model structure.

**Figure 10:**
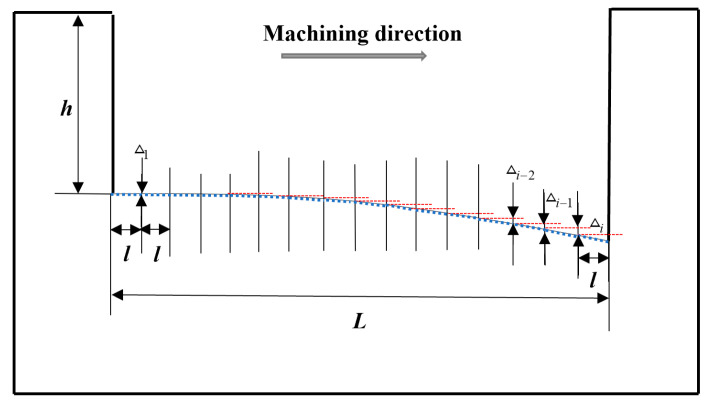
Microscale theoretical electrode compensation path diagram.

After a comparative analysis, the power function is selected for nonlinear fit, and finally, the theoretical electrode compensation path equation is obtained as $g(x)=0.0862e^{0.0046x}$. Because the machined tool electrode needs to adopt the interpolation principle in the actual machining process, the theoretical electrode compensation path needs to be simplified. The whole *g*(*x*) curve, after micro-segmentation, can be regarded as a straight line with a changing slope change for each segment (*l*), which is the theoretical electrode compensation for each segment (*l*). As shown in blue dotted line in Figure 10, which simplifies the theoretical electrode compensation path.

### 4.3. Compensation Machining

**Figure 11:**
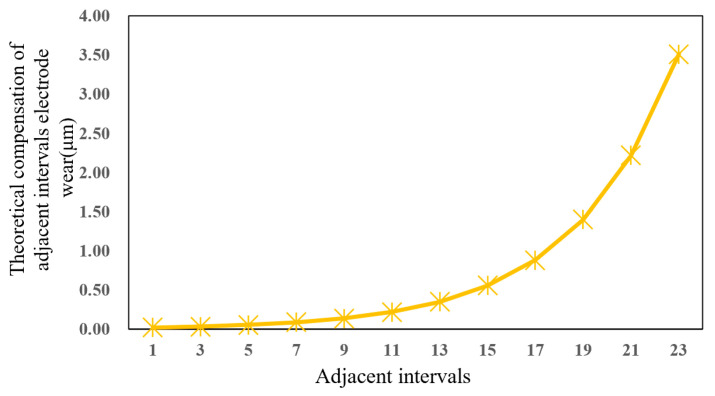
Theoretical compensation of adjacent intervals electrode wear.

In this paper, a fixed length of *l* = 50 μm is selected. Under the *GRA* optimization parameters, the feed depth *H* = 600 μm is set, and microgroove micro-EDM with compensation is carried out for machining length of *L* = 1000 μm and *L* = 1200 μm, respectively. The microgroove length *L* = 1200 μm is divided into 24 equal parts, and the theoretical electrode wear compensation equation is obtained to calculate the theoretical electrode compensation for adjacent intervals, as shown in Figure 11.

The ANN model predicts the electrode axial wear for each machining length under the theoretical compensation path, and the electrode axial wear △z between adjacent lengths is recorded in Table 8. Thus, the actual electrode compensation in the microgroove micro-EDM process can be obtained.

**Figure 12:**
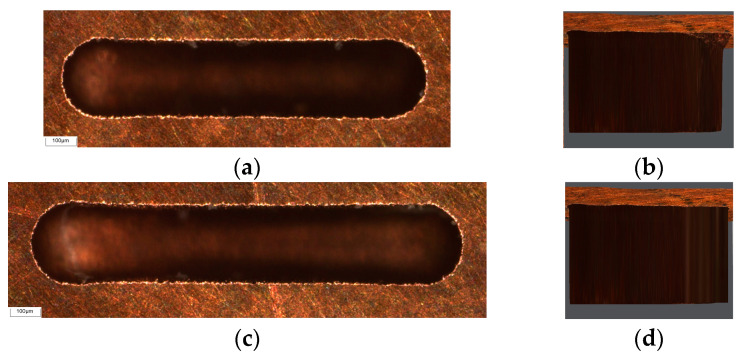
Microgrooves morphology and profile with compensation. (**a**) L = 1000 μm compensated microgroove morphology; (**b**) L = 1000 μm compensated microgroove profile; (**c**) L = 1200 μm compensated microgroove morphology; (**d**) L = 1200 μm compensated microgroove profile.

The ultra-depth of field optical microscope is used to analyze the morphology and bottom profile of the microgroove with compensation machining. As shown in Figure 12, it can be seen that the width and depth of the machined microgrooves before and after machining are highly consistent, indicating that the fixed-length nonlinear compensation method has a certain compensation accuracy and feasibility, which improves the quality of the microgroove micro-EDM process to a certain extent.

**Figure 13:**
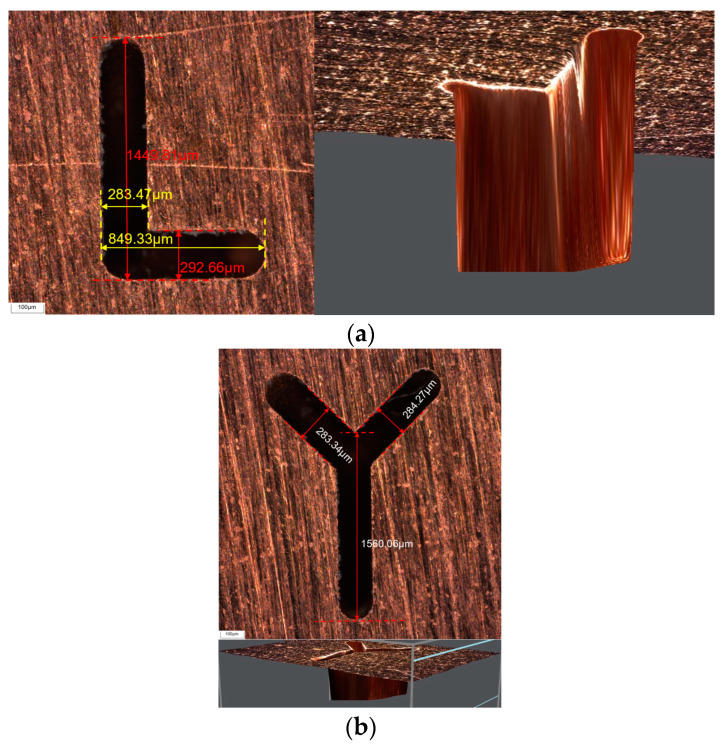
Special-shaped microgroove structure. (**a**) L shape microgroove with H = 600 μm (**b**) Y shape microgroove with H = 100 μm.

Using the proposed compensation method, the special-shaped groove structure is processed with compensation, as shown in Figure 13.

## 5. Conclusions

1. Through orthogonal experiment design and analysis, the influence of machining parameters on machining performance can be obtained. The influence of *I_p_*, *U_p_*, and *f_p_* on machining time is significantly greater than that of *w_p_* and *Rot*. The influence of the non-electrical factor *Rot* on electrode axial and radial wear is significantly smaller than that of the electrical factors *I_p_*, *U_p_*, *f_p_* and *w_p_*.
2. *The GRA* method is used to optimize machining performance at the same time. The optimized combination of factor levels is *I_p_* = 30 Index, *U_p_* = 100 V, *f_p_* = 120 kHz, *w_p_* = 4 μs, and *Rot* = 700 rpm. The machining time required for microgroove micro-EDM is 50.28 s, electrode axial wear is 152.29 μm, and electrode radial wear is 55.52 μm. Compared to the H17 orthogonal experiment, the machining time is shortened by 10.01%, electrode axial wear is reduced by 2.02%, and electrode radial wear is reduced by 10.80%.
3. Compared to microgroove machined without compensation under the GRA optimization factors combination, the fixed-length nonlinear compensation method proposed in this paper is used to compensate for electrode axial wear during machining. As a result, microgroove morphology with good consistency of depth and width is machined, which improves the machining quality of microgroove. It also shows that the compensation method has certain compensation accuracy and feasibility.

## Figures and Tables

**Table 1:** Physical properties of copper.

Density (g/cm^3^)	Melting Point (°C)	Poisson’s Ratio	Specific Heat Capacity (J/kg×K)	Modulus of Elasticity (GPa)	Thermal Conductivity (W/m×K)
8.96	1083	0.33	385	100–110	401

**Table 2:** Physical properties of EDM oil.

Dielectric Constant	Insulation Strength (mv/m)	Density (Kg/m^3^)	Stickiness (mm^2^/s)	Combustion Point (°C)	Flash Point (°C)
3	14–22	813	7.0	243	134

**Table 3:** Experiment parameters and level.

Level	*I_p_* (A)	*U_p_* (V)	*f_p_* (kHz)	*Rot* (rpm)	*w_p_* (μs)
1	25	90	110	600	3
2	30	100	120	700	4
3	35	110	130	800	5

**Table 4:** L27(3^5^) orthogonal experiment.

Number	*I_p_* (A)	*U_p_* (V)	*f_p_* (kHz)	*Rot* (rpm)	*w_p_* (μs)
H1	1(25)	1(100)	1(110)	1(600)	1(2)
H2	1	1	1	1	2(3)
H3	1	1	1	1	3(4)
H4	1	2(110)	2(120)	2(700)	1
H5	1	2	2	2	2
H6	1	2	2	2	3
H7	1	3(120)	3(130)	3(800)	1
H8	1	3	3	3	2
H9	1	3	3	3	3
H10	2(30)	1	2	3	1
H11	2	1	2	3	2
H12	2	1	2	3	3
H13	2	2	3	1	1
H14	2	2	3	1	2
H15	2	2	3	1	3
H16	2	3	1	2	1
H17	2	3	1	2	2
H18	2	3	1	2	3
H19	3(35)	1	3	2	1
H20	3	1	3	2	2
H21	3	1	3	2	3
H22	3	2	1	3	1
H23	3	2	1	3	2
H24	3	2	1	3	3
H25	3	3	2	1	1
H26	3	3	2	1	2
H27	3	3	2	1	3

**Table 5:** Grey relational grade of each performance indicator.

Number	Machining Time	Axial Electrode Wear	Radial Electrode Wear	Grey Relational Grade	Rank
1	0.683774	0.355223	0.422137	0.487045	26
2	0.535804	0.410972	0.465554	0.470777	27
3	0.7	0.504785	0.46019	0.554991	22
4	0.620897	0.618066	0.605628	0.614864	12
5	0.565995	0.78091	0.733858	0.693588	2
6	0.494982	0.777784	0.667543	0.64677	5
7	0.601902	0.549356	0.733333	0.628197	6
8	0.451408	0.549321	0.5	0.500243	25
9	0.617012	0.502239	0.50976	0.543004	24
10	0.582181	0.673715	0.612959	0.622952	9
11	0.530232	0.780498	0.566058	0.625596	7
12	0.564363	0.870667	0.534302	0.656444	4
13	0.567464	0.683463	0.613082	0.621336	10
14	0.666667	0.636953	0.568709	0.624109	8
15	0.63688	0.584366	0.641194	0.620813	11
16	0.58254	0.729469	0.666069	0.65936	3
17	0.557034	0.81637	0.750316	0.707907	1
18	0.564913	0.645772	0.625597	0.612094	13
19	0.531063	0.632498	0.559871	0.574477	19
20	0.509877	0.590246	0.615013	0.571712	20
21	0.563142	0.554741	0.6447	0.587528	18
22	0.60052	0.647126	0.578038	0.608561	14
23	0.53989	0.614634	0.648712	0.601079	16
24	0.560445	0.547922	0.592146	0.566837	21
25	0.622941	0.625676	0.569308	0.605975	15
26	0.554208	0.584631	0.656491	0.598443	17
27	0.528305	0.549103	0.572383	0.54993	23

**Table 6:** Grey relational grade of each machining parameter and level.

Level	*I_p_*	*U_p_*	*f_p_*	*w_p_*	*Rot*
1	0.567866	0.576936	0.557011	0.574984	0.634855
2	0.715842	0.698822	0.72796	0.635448	0.718579
3	0.496075	0.520651	0.509786	0.596781	0.530551
Range	0.219768	0.178171	0.218173	0.060464	0.188028

**Table 7:** The fitted goodness of different nonlinear functions.

Nonlinear Functions	*R* ^2^	*Adj R* ^2^	*RMSE*
$g(x)=ae^{bx}$	0.9943	0.9924	0.0080
$g(x)=ax^{b}$	0.9719	0.9625	1.5742
$g(x)=ax^{2}+bx+c$	0.9853	0.9706	1.3936

**Table 8:** Adjacent interval electrode compensation.

Number of Intervals	Theoretical Compensation (μm)	Electrode Wear During Compensation (μm)	Actual Compensation (μm)
1	0.0223	0.0045	0.0268
3	0.0354	0.0071	0.0425
5	0.0559	0.0112	0.0671
7	0.0886	0.0230	0.1116
9	0.1403	0.0365	0.1768
11	0.2223	0.0667	0.2890
13	0.3522	0.1127	0.4649
15	0.5579	0.1897	0.7476
17	0.8838	0.3182	1.2020
19	1.4000	0.5880	1.9880
21	2.2177	0.9980	3.2157
23	3.5129	1.6862	5.1991

## Data Availability

Data will be made available on request.

## Abbreviations

The following abbreviations are used in this manuscript:

EDM	Electric discharge machining
ANN	Artificial neural network

## References

[1] Wang P., Bai Q. (2022). The modelling and analysis of micro-milling forces for fabricating thin-walled micro-parts considering machining dynamics. Machines.

[2] Lin W., Fan F. (2022). Analysis of the warpage phenomenon of micro-sized parts with precision injection molding by experiment, numerical simulation, and grey theory. Polymers.

[3] Mouralova K., Bednar J. (2022). Production of precision slots in copper foil using micro EDM. Sci. Rep..

[4] Zhang H., Wang X. (2021). Formability and mechanism of pulsed current pretreatment-assisted laser impact micro-forming. J. Adv. Manuf. Technol..

[5] Hu Y., Qi H. (2023). Topologically devised flexible bi-aeolotropic conduction Janus-like bi-layer membrane functionalized by red-green bicolor fluorescence. J. Mater. Res. Technol..

[6] Wang J., Yang M. (2024). Ventilation condition effects on heat dissipation of the lithium-ion battery energy storage cabin fire. Case Stud. Therm. Eng..

[7] Anwar S., Rasool G. (2024). Impact of Viscous Dissipation and Ohmic Heating on Natural Convection Heat Transfer in Thermo-Magneto Generated Plume. Front. Heat Mass Transf..

[8] Zhang S., Shi H. (2024). Research on the Milling Performance of Micro-Groove Ball End Mills for Titanium Alloys. Lubricants.

[9] Cao Z.-H., Tang J. (2025). Full Cross-Sectional Profile Measurement of a High-Aspect-Ratio Micro-Groove Using a Deflection Probe Measuring System. Sensors.

[10] Peng Z., Feng T. (2019). Directly writing patterning of conductive material by high voltage induced weak electric arc machining (HV-μEAM). Coatings.

[11] Zhuang W.H. (2021). Research on Microfine EDM Method for Strip Electrodes with Microchannel Structure.

[12] Bissacco G., Tristo G. (2021). Uncertainty of the electrode wear on-machine measurements in micro-EDM milling. J. Manuf. Process..

[13] Mahbub M.R., Rashid A. (2021). Strategies of improving accuracy in micro-EDM. Micro Electro-Fabrication.

[14] Tong H., Li Y. (2018). Servo scanning 3D micro EDM for array micro cavities using on-machine fabricated tool electrodes. J. Micromechan. Microeng..

[15] Sharma A. (2006). Zero Wear Electrode for EDM Using Electrically Conductive Diamond. Ph.D. Thesis.

[16] Yu Z.Y., Masuzawa T. (1998). Micro-EDM for three-dimensional cavities-development of uniform wear method. CIRP Ann..

[17] Aliyu A.A., Rohani J.M. (2017). Optimization of electrical discharge machining parameters of Si/SiC through response surface methodology. J. Technol..

[18] Kaneko T., Tsuchiya M. Improvement of 3D NC contouring EDM using cylindrical electrode-optical measurement of electrode deformation and machining of free-curves. Proceedings of the 10th International Symposium for Electro Machining (ISEM-X).

[19] Yu Z.Y., Rajurkar K.P. (2004). Study of 3D micro-ultrasonic machining. J. Manuf. Sci. Eng..

[20] Yu Z.Y., Kozak J. (2003). Modelling and simulation of micro EDM process. CIRP Ann..

[21] Hang G.R., Cao G.H. Micro-EDM milling of micro platinum hemisphere. Proceedings of the 1st IEEE International Conference on Nano/Micro Engineered and Molecular Systems.

[22] Gou J., Lai J. (2022). Fabrication of high accuracy micro-hole by micro-EDM under tool electrode spiral motion feed mode aided with fixed reference axial compensation. Res. Sq..

[23] Karmiris P. (2025). Multiphysics Thermo-fluid Modeling and Experimental Validation of Crater Formation and Rim Development in EDM of Inconel C-276. Simul. Model. Pract. Theory.

[24] Zhong M., Yu H. (2025). Optimization of heat treatment process parameters for GH4738 superalloy based on Taguchi-Grey relational analysis method. Mater. Lett..

[25] Winarso R., Slamet S. (2025). Optimization of 3D Printed Voronoi Microarchitecture Bone Scaffold using Taguchi-Grey Relational Analysis. Bioprinting.

[26] Xu J.K., Li M.Y. (2022). Process Parameter Modeling and Multi-Response Optimization of Wire Electrical Discharge Machining NiTi Shape Memory Alloy. Mater. Today Commun..

[27] Richard S.P., Titus S. (2025). Efficient energy management using Red Tailed Hawk optimized ANN for PV, battery & super capacitor driven electric vehicle. J. Energy Storage.

[28] Wang Y.Z. (2021). Detection of lane line based on Robert operator. JME.

[29] Wang Y., Yin T. (2024). A steel defect detection method based on edge feature extraction via the Sobel operator. Sci. Rep..

[30] Tenekeci M., Abdulazeez S.T. (2024). Edge detection using the Prewitt operator with fractional order telegraph partial differential equations (PreFOTPDE). Multimedia Tools and Applications.

[31] Su Z.Y., Zhang W.H. (2024). Quantum image edge detection based on Laplacian of Gaussian operator. Quantum Inf. Process..

[32] Ma P., Yuan H. (2024). A Laplace operator-based active contour model with improved image edge detection performance. Digit. Signal Process..

[33] Gaurak, Ghanekar U. (2018). Image steganography based on canny edge detection, dilation operator and hybrid coding. J. Inf. Secur. Appl..

[34] Zhang M., Guo H. (2023). Compensation method of wire electrode wear for reciprocating micro wire electrical discharge machining. J. Manuf. Process..

[35] Zhang X.D., Li Y.Q. (2024). High-quality and efficiency machining of micro-EDM. Proceedings of the 2024 IEEE International Conference on Manipulation, Manufacturing and Measurement on Nanoscale (3M NANO).

